# VIRMOTIF: A User-Friendly Tool for Viral Sequence Analysis

**DOI:** 10.3390/genes12020186

**Published:** 2021-01-27

**Authors:** Pedram Rajaei, Khadijeh Hoda Jahanian, Amin Beheshti, Shahab S. Band, Abdollah Dehzangi, Hamid Alinejad-Rokny

**Affiliations:** 1 Amirkabir University of Technology, Tehran 346512, Iran; pedram56rajaii@gmail.com; 2 AI-enabled Processes (AIP) Research Centre, Health Data Analytics Program, Macquarie University, Sydney, NSW 2109, Australia; hoda.jahanian@gmail.com; 3 Department of Computing, Macquarie University, Sydney, NSW 2109, Australia; 4 Future Technology Research Center, College of Future, National Yunlin University of Science and Technology, 123 University Road, Yunlin 64002, Taiwan; shamshirbands@yuntech.edu.tw; 5 Department of Computer Science, Rutgers University, Camden, NJ 08102, USA; i.dehzangi@rutgers.edu; 6 Center for Computational and Integrative Biology, Rutgers University, Camden, NJ 08102, USA; 7 Biological & Medical Machine Learning Lab (BML), The Graduate School of Biomedical Engineering, The University of New South Wales, UNSW, Sydney, NSW 2052, Australia

**Keywords:** sequence analysis, motif analysis, D-ratio, virus genome, sequence variation

## Abstract

Bioinformatics and computational biology have significantly contributed to the generation of vast and important knowledge that can lead to great improvements and advancements in biology and its related fields. Over the past three decades, a wide range of tools and methods have been developed and proposed to enhance performance, diagnosis, and throughput while maintaining feasibility and convenience for users. Here, we propose a new user-friendly comprehensive tool called VIRMOTIF to analyze DNA sequences. VIRMOTIF brings different tools together as one package so that users can perform their analysis as a whole and in one place. VIRMOTIF is able to complete different tasks, including computing the number or probability of motifs appearing in DNA sequences, visualizing data using the matplotlib and heatmap libraries, and clustering data using four different methods, namely K-means, PCA, Mean Shift, and ClusterMap. VIRMOTIF is the only tool with the ability to analyze genomic motifs based on their frequency and representation (D-ratio) in a virus genome.

## 1. Introduction

According to bioinformatics terminology, sequence analysis is defined as the process of applying analytical methods to DNA, RNA, or protein sequences to retrieve meaningful information. Among the sequence analysis methods, sequence alignment and similarity analysis are widely used to retrieve and present information. The main goal of sequence alignment and similarity analysis is to show similarities and differences among biological sequences.

With the exponential increase in available biological sequences in the world, the importance of fast and accurate sequence analysis tools and packages is also increasing. Currently, a comprehensive package or tool to analyze sequence variability in viral genomes does not exist. Hence, there is a demand for comprehensive tools and packages that enable users to conduct several tasks such as motif analysis, motif frequency visualization, and sequence clustering together and in one place.

Variation in viral genomic motifs is typically detected by aligning the sequences and comparing the query sequences to a reference sequence. Commonly, multiple alignment needs to be performed to provide a consensus sequence that can be used as a reference sequence. This consensus/reference sequence might not be the original ancestral sequence of the query sequences. This approach has been used widely in order to identify virus genomic patterns such as vaccine targets [1,2,3,4] and also in the analysis of cancer mutational profiles [5,6]. Using alignment to analyze genomic motifs is very useful when a parental reference sequence is available. However, when a parental sequence is not available (e.g., in individuals infected with natural HIV and/or SIV), using an alignment method may not be suitable to analyze genomic motifs. Therefore, it is important to use a method that can identify and quantify genomic motifs without needing the alignment of a reference sequence. In this case, machine learning techniques have been used widely in order to analyze motifs in human and viral genomes [7,8,9,10,11,12,13]. Among the machine learning methods, studies have shown that Markov models of conditional probabilities can be used as a more accurate estimation of motif representation in human and viral genomes [14,15]. In these methods, the expected frequency of a motif is estimated using the observed frequencies of the motif constituents, considering the overlapping nucleotide(s).

In this paper, we propose a new tool called VIRMOTIF, which undertakes different tasks related to sequence analysis. Given one or more DNA sequences, VIRMOTIF is able to analyze their k-mer motifs using the frequency representation of genomic motifs [15,16]. A k-mer motif is a genomic motif, in which k can be 1, 2, 3,..., N. VIRMOTIF is also able to analyze viral sequences using the D-ratio representation of k-mer motifs. In this method, the expected frequency of a motif is estimated using the observed frequencies of the motif constituents, considering the overlapping nucleotide(s) [15,16]. VIRMOTIF is also able to cluster and visualize viral sequences. VIRMOTIF conducts clustering tasks using four different algorithms namely, K-means, Principal Component Analysis (PCA), Mean Shift, and ClusterMap. In addition, a feature of VIRMOTIF is its ability to visualize results by incorporating matplotlib as a powerful visualization library.

## 2. Materials and Methods

VIRMOTIF is able to undertake different tasks related to sequence analysis. Each of those tasks is presented and explained below.

### 2.1. Motif Analysis Based on Frequency

VIRMOTIF is able to conduct sequence analysis based on the frequency of its motifs. Assume that there are several DNA sequences and we want to find the frequency of their k-mer motifs to compare and analyze the sequences. To do this, VIRMOTIF first computes the frequency of the k-mer motifs by observing each sequence, separately. It then finds all the substrings with a maximum length of 5 and, finally, calculates their frequencies for all the given DNA sequences, separately. Note that as the maximum length of substrings increases, the number of generated motifs increases exponentially. Hence, to avoid generating an enormous number of motifs, we confined the maximum length to 5-mers. VIRMOTIF then normalized the data by dividing each frequency number by the number of k-mer motifs.

### 2.2. Motif Analysis Based on the Markov Model (D-Ratio)

VIRMOTIF is also able to conduct motif analysis based on the Markov model (D-ratio) [15,16]. To conduct this analysis, we need a clear definition of the “representation” concept. In this study, “representation” is defined as the ratio of the observed frequency (P_obs_) of a motif over its expected frequency (P_exp_) in the genome. The Pobs of a motif is defined as the number of times that motif appears in the sequence divided by the total number of all motifs with the same length. P_exp_ can be calculated differently, as shown below:

(1) $$P_{\exp}\left(\mathrm{CpGpT}\right)=P_{\mathrm{obs}}\left(C\right)\times P_{\mathrm{obs}}\left(G\right)\times P_{\mathrm{obs}}\left(T\right)$$

(2) $$=P_{\mathrm{obs}}\left(\mathrm{CpG}\right)\times P_{\mathrm{obs}}\left(T\right)$$

(3) $$=P_{\mathrm{obs}}\left(C\right)\times P_{\mathrm{obs}}\left(\mathrm{GpT}\right)$$

In this study, we used first- and second-order models to calculate the expected frequencies of trinucleotides and tetranucleotides, respectively, as was done in [15]. Examples of first- and second-order Markov models are given in Equations (4) and (5).

(4) $$P_{\exp}\left(\mathrm{CpGpT}\right)=\frac{P_{\mathrm{obs}}\left(\mathrm{CpG}\right)\;\times {\;P}_{\mathrm{obs}}\left(\mathrm{GpT}\right)}{P_{\mathrm{obs}}\left(G\right)}$$

(5) $$P_{\exp}\left(\mathrm{CpGpTpA}\right)=\frac{P_{\mathrm{obs}}\left(\mathrm{CpGpT}\right)\;\times {\;P}_{\mathrm{obs}}\left(\mathrm{GpTpA}\right)}{P_{\mathrm{obs}}\left(\mathrm{GpT}\right)}$$

We then calculated the representation (D-ratio) for each motif by dividing its P_obs_ by its P_exp_. This is shown in Equation (6), using the TGGG motif as an example. The expected probability of the tetranucleotide motif TGGG was computed using the observed probabilities of its dinucleotides (TG, GG) and mononucleotide G constituents.

(6) $$D\left(\mathrm{TGGG}\right)=\frac{p_{\mathrm{obs}}\left(\mathrm{TGGG}\right)}{p_{\exp}\left(\mathrm{TGGG}\right)}=\frac{p_{\mathrm{obs}}\left(\mathrm{TGGG}\right)}{\frac{p_{\mathrm{obs}}\left(\mathrm{TGG}\right)\times p_{\mathrm{obs}}\left(\mathrm{GGG}\right)}{p_{\mathrm{obs}}\left(\mathrm{GG}\right)}}.\;$$

The computed D-ratio in Equation (6) is a pure “representation” of TGGG, which, importantly, is independent of the changes that may happen in TGG, GGG, and GG. Using the Markov model, we produced a large number of features to present our data. It has been shown that when there is a large number of variables in the data (i.e., high-dimensional datasets such as biological datasets), extracting information from the data can be difficult [15,16]. Therefore, it is necessary to reduce the number of variables without losing useful information. Here, we employed the Principal Component Analysis (PCA) model to reduce the dimension of data and extract informative features. PCA is explained in detail in the following subsection.

### 2.3. Clustering of Sequences

Clustering of sequences is the process of bringing similar motifs together as a cluster. Different clustering approaches have been previously used to group biological and nonbiological data [17,18,19,20]. Clustering of sequences gives the user the ability to observe each group separately, identify similar patterns among samples in the same group, identify differences between different clusters, and find the most important motifs in each group. Here, we implemented four widely used clustering methods in VIRMOTIF, namely K-means, PCA with K-means, Mean Shift, and ClusterMap.

### 2.4. K-Means

K-means is one of the most popular clustering methods [21]. K-means divides the dataset into K groups, in which each group has a center and data are grouped if and only if their distance to the group center is smaller compared to other centers. In K-means, the user specifies a subset of motifs and the number of clusters (K). Therefore, this method is mainly used in cases when the number of clusters is known [21]. This is particularly useful when the user has some information about the nature of their data (e.g., they know how many viruses are going to be analyzed). Similar to K-means, in PCA, the user also specifies a subset of k-mer motifs and the number of clusters [22]. In this study, PCA is mainly incorporated and used (which is explained in the next subsection) for dimension reduction and the normalization of the dataset [22].

### 2.5. Mean Shift

VIRMOTIF also uses Mean Shift for clustering. Mean Shift is able to set the number of clusters automatically [23]. The Mean Shift method works in two phases. In the first phase, the optimal number of clusters is determined. To do this, Mean Shift uses a probability density function from which the data is drawn then, via kernel density estimation techniques, puts centroids of clusters at the maximal level of that density function. In the second phase, Mean Shift clusters the data using the number of clusters identified in the first phase. In the Mean Shift method, the user only needs to choose the subset of k-mer motifs and Mean Shift will do the rest. VIRMOTIF uses clustering methods that are implemented in the scikit-learn package to cluster the sequences.

### 2.6. Principal Component Analysis (PCA)

PCA is an orthogonal linear transformation to convert a dataset to a lower dimension [22]. The two greatest variances by the scalar projection are named first- and second-principal components, respectively. We can use these principal components to extract features from data [22]. For normalization purposes, we find the maximum absolute value in the dataset and divide the whole dataset by this number. This process scales the dataset to an interval of (−1,1). Here, we use PCA and K-means together because after applying dimensional reduction and normalization, the subspace spanning the principal direction is the same compared to the cluster centroid subspace [24,25,26,27]. For example, the following figure is an example of PCA analysis using VIRMOTIF on 3000 hepatitis B viruses.

### 2.7. ClusterMap

Cluster mapping is a method for calculating the hierarchical structure of data. It uses a minimum spanning tree algorithm with a similarity/distance function (e.g., the Euclidian metric) and finally reorders data to groups of stacked rectangles. It assigns a color to each rectangle based on their values. A larger difference in values indicates a larger difference in colors.

### 2.8. Visualization of Data

VIRMOTIF is also able to visualize the results as well as its produced output in a user-friendly and informative manner. The purpose of visualization in VIRMOTIF is to enable users to observe and detect patterns of motifs much easier and more efficiently from clustered data or a raw dataset. VIRMOTIF also enables the user to plot the output of clustering methods.

### 2.9. Bar Chart Visualization

Here, we use matplotlib, which is a python package for visualization. To do this, VIRMOTIF enables the user to select between frequency or D-ratio for the basis of computation. Users can choose a subset of motifs and plot their 3D bar chart.

### 2.10. Heatmap Visualization

One of the well-known techniques for data visualization is heatmap visualization [28]. Sequence analysis uses the ratio between data and shows it as a titling rectangle with a different range of colors. In VIRMOTIF, the user has access to this feature in the Plot section of the tool.

## 3. How to Use VIRMOTIF

**Figure 1:**
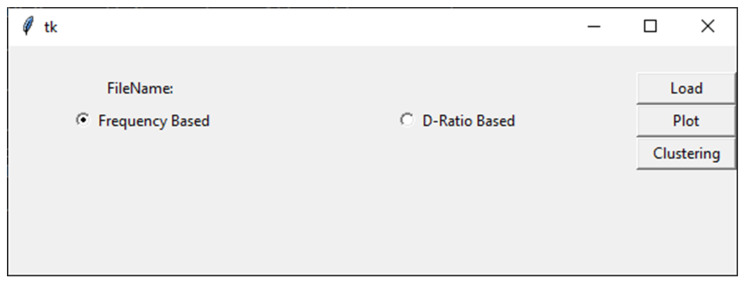
Overview of the VIRMOTIF main page.

As mentioned earlier, VIRMOTIF is a tool developed for sequence analysis, which allows the user to visualize and cluster datasets. When the user opens the application, there are three different sections, as shown in Figure 1. There is a section for loading datasets. Here, the user can upload files to VIRMOTIF by clicking on the load button. Upon uploading, the number of motifs is computed by the frequency and D-ratio; the user is then able to choose between them. The name of the file is shown on the top of the interface. Another section is to cluster and plot the data. Here, the user can click on the Plot button or the Clustering button to open their respective interfaces. Below is an instance of the main page (Figure 1).

**Figure 2:**
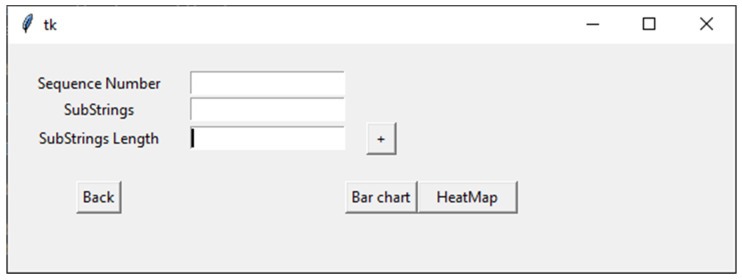
An overview of the plotting options in VIRMOTIF.

When the user clicks on the Plot button, the plotting interface opens, which contains three text boxes (as shown in Figure 2). Here, the first text box enables inputting the number of sequences that are going to be plotted. In the second text box, the user can choose motifs by naming the motifs, writing the length of motifs, or picking the length of the motifs with the “+” action button next to the third text box. The user can then begin plotting using either a bar chart or heatmap.

**Figure 3:**
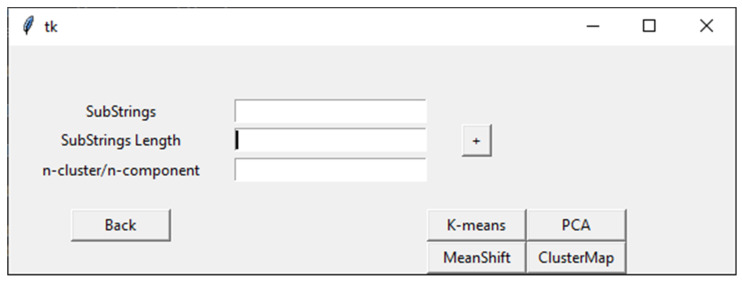
An overview of the Clustering page in VIRMOTIF.

When the user selects the Clustering action button in the VIRMOTIF homepage (Figure 1), the clustering page opens (as shown in Figure 3). There are four sections in this page. In addition to the three sections in the Plot page, there is a section for the user to choose the method for clustering, which are K-means, Mean Shift, ClusterMap, and PCA. These models and their tasks are explained in detail in the Materials and Methods section (Section 2).

**Figure 4:**
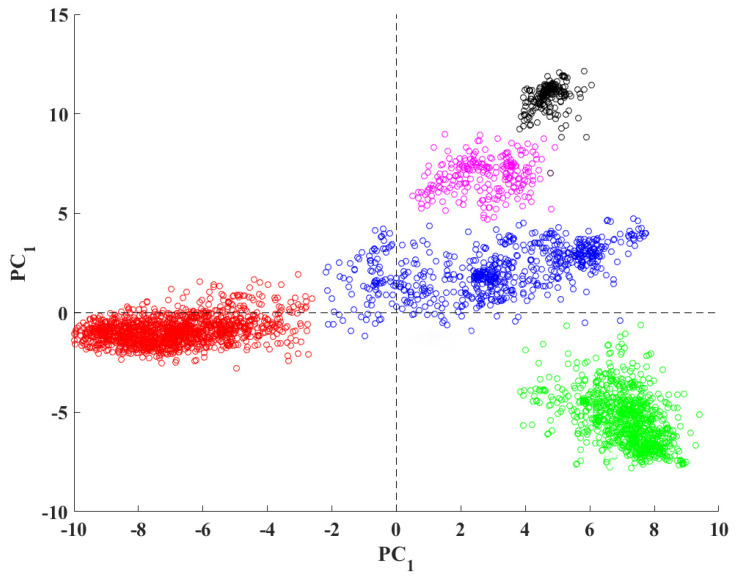
PCA analysis of the D-ratio of the 96 3-mer motifs of 3000 hepatitis B virus (HBV) sequences, calculated by VIRMOTIF.

Here, we present several examples to provide more information on how VIRMOTIF works. To do this, we used ~3000 whole-genome sequences of hepatitis B virus (HBV) downloaded from the National Center for Biotechnology Information (NCBI). We performed PCA, bar chart visualization, and Cluster Map analysis as some examples of running VIRMOTIF on a virus genome dataset. Figure 4 shows application of PCA feature of VIRMOTIF on the HBV sequencers, resulting in 5 clusters.

**Figure 5:**
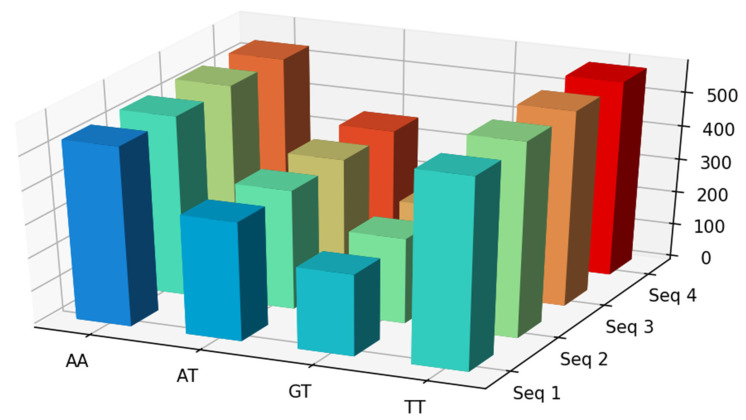
Visualization of first four sequences with substrings AA, AT, GT, and TT. In this example, we used frequencies as the computation method.

Below is an example of the visualization of four HBV sequences with substrings AA, AT, GT, and TT (Figure 5).

**Figure 6:**
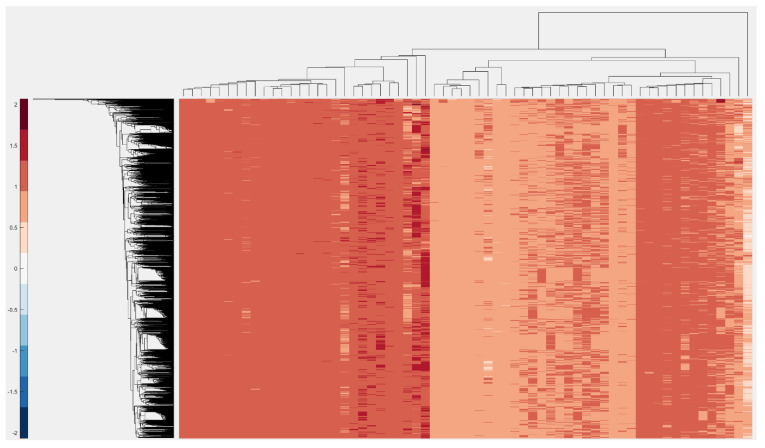
Here, we selected the D-ratio for computing the motifs and substrings with length of 2 and the Cluster Map method to cluster the data.

Another example of clustering can be seen below in Figure 6. Here, we used the D-ratio of 96 3-mers sequences in 3000 HBV sequences and then performed a clustered heatmap analysis using the “clustermap” feature of VIRMOTIF.

## 4. Conclusions

We developed VIRMOTIF, a user-friendly bioinformatics tool, which is able to conduct different tasks to help users explore different groups of motifs, conveniently. This tool can be used to identify genomic motifs that are more likely to have a significant effect on a wide range of infectious diseases, using this information to discover new treatments. VIRMOTIF is easy to expand and new features, such as new types of clustering algorithms, can be added to it based on the users’ needs. In the future, we aim at investigating larger motifs and increase the maximum length. We also aim at incorporating a larger number of feature reduction and clustering methods such as forward or backward selections (for feature reduction) and hierarchical model (for clustering). VIRMOTIF is publicly available at: https://gitlab.com/pedram56rajaii/virmotif [29].

## Data Availability

Data available in a publicly accessible repository. The data presented in this study are openly available through NCBI repository. The VIRMOTIF Python package along with its detailed user manual are publicly available at https://gitlab.com/pedram56rajaii/virmotif.
